# Interventions of a clinical pharmacist in a medical intensive care unit – A retrospective analysis

**DOI:** 10.17305/bjbms.2020.4612

**Published:** 2020-11

**Authors:** Maja Cvikl, Andreja Sinkovič

**Affiliations:** 1 Central Pharmacy, University Medical Centre Maribor, Maribor, Slovenia; 2 Department of Medical Intensive Care, University Medical Centre Maribor, Maribor, Slovenia

**Keywords:** Clinical pharmacy, intensive care, interventions, medication errors, multidisciplinary care, Slovenia

## Abstract

Several studies demonstrated a significant decrease in prescription errors, adverse drug events, treatment costs and improved patient outcomes, when a clinical pharmacist (CP) was a full member of a multidisciplinary team in the intensive care unit (ICU). Our aim was to evaluate the activities of a CP, included in a 12-bed medical ICU team of a university hospital in the course of several months. We conducted a retrospective analysis of all the CP’s interventions from March 2017 to November 2017, carried out and documented after reviewing and discussing patients’ medical data with the treating ICU physicians. We identified four main categories of CP’s interventions: pharmacotherapy adjustments to kidney function (PAKF category), drug-drug interactions (DDIs category), therapeutic monitoring of drugs with narrow therapeutic index (TDM category), and drug administration by the nasogastric tube (NGT category). During the study period, 533 patients were admitted to the medical ICU. The CP reviewed the medical data of 321 patients and suggested 307 interventions in 95 patients. There were 147 interventions of the PAKF category, 57 interventions of the TDM category, 30 interventions of the NGT category, and 22 interventions of the DDIs category. Fifty-one interventions were unspecified. The majority of all interventions (203/307) were related to antimicrobial drugs. ICU physicians completely accepted 80.2% of the CP’s suggestions. We observed that regular participation of the CP in the medical ICU team contributed to more individualized and improved pharmacological treatment of patients. Therefore, ICU teams should be encouraged to include CPs as regular team members.

## INTRODUCTION

Several studies demonstrated a significant role of a clinical pharmacist (CP) as a full member of a multidisciplinary team in the intensive care unit (ICU) [1-5]. Full membership of a CP in the ICU team includes the presence at the daily rounds in the ICU, cooperation, and availability for consultations throughout the day [6].

CPs in ICUs are focused on the optimal use of pharmacotherapy to improve patients’ safety and to achieve increased survival as well. The competences of CPs in the critical care settings are of significant importance due to the patient’s critical condition, polypharmacy, and rapid and frequent modifications of pharmacotherapy. Correct drug dosing, identification and prevention of adverse effects, drug interactions, and errors in pharmacotherapy are among the most important goals for CPs. CPs should also suggest alternative therapies, cost-effective use of drugs and provide education of ICU staff, in particular in the use of antibiotics. It is not surprising that the number of CPs throughout the world has increased in recent decades and their role in many health-care teams became well defined [7,8]. In addition, the Critical Care Societies consider a CP as an essential member of the critical care team [9]. In developed countries, pharmacists can now be found in almost all critical care units [8].

Our aim was to evaluate the activities of a CP, recently included as a part-time member of a medical ICU team in a university clinical center during a period of several months.

## MATERIALS AND METHODS

We conducted a retrospective analysis of the CP’s interventions from March 1 to November 30, 2017, in a 12-bed adult medical ICU at University Medical Centre Maribor. The CP became a regular part-time member of the medical ICU team shortly before March 2017. Before regular involvement with the ICU team, the CP was trained by an experienced senior CP for several months and was further gaining experience in clinical ICU pharmacy during 5-year consultations at the request of the attending ICU physician.

All patients admitted to the medical ICU over the study period were eligible for inclusion, and patients with at least one prescription reviewed by the CP were included in the study.

During the study period, the CP visited the medical ICU every working day (Monday–Friday) from 11 a.m. to 1 p.m. (excluding weekends and holidays), checking the diagnosis of each patient, daily laboratory and microbiology test results, and the prescribed therapy of each patient.

The CP suggestions of drug dosing regimen were based on multiple literature sources: the Summary of product characteristics (SmPC) of every specific drug [10], UpToDate^®^ Database [11], Micromedex^®^ Database [12], The Renal Drug Handbook [13], Critical Care Pharmacotherapy [14], and others [15,16].

The CP daily assessed the route of administration for the prescribed therapy - subcutaneous (sc.), intravenous (iv.), oral, or by a nasogastric tube (NGT). If a drug was administered by a NGT, the CP suggested a different dosage form of the same active substance or alternative medication, if originally prescribed medication was unsuitable [10,17].

For the daily review of the prescribed therapy for drug-drug interactions (DDIs), the CP used mainly Lexicomp^®^ interaction database and considered only clinically relevant interaction of type D (consider therapy modification) and type X (avoid combinations). For specific interactions, the CP studied in detail case-report and case-series reviews (e.g., fentanyl – linezolid interaction [18-20], esomeprazole – clopidogrel interaction [21-25], and carbapenem – valproate interaction [26-30]).

Pharmacist-guided therapeutic drug monitoring (TDM) for vancomycin, gentamicin, and amikacin has already been established at our university hospital years before, using the pharmacokinetic program Kinetidex^®^. The TDM service was continued by the CP in the ICU team.

The CP actively participated in the department’s daily main round and discussed every suggestion or comment with the treating ICU physician and other members of the ICU team. The CP documented acceptance of suggestions.

Retrospectively, the CP’s interventions were sorted into five categories: i) pharmacotherapy adjustments to kidney function (PAKF category) [mainly adjustments of dosing regimen], ii) TDM category (dosing adjustments according to serum concentration of drugs with a narrow therapeutic index), iii) DDI category, iv) administration of drugs through the NGT (NGT category), and v) unspecified interventions (Unspecified category). Acceptance of suggestions was classified either as “completely accepted” (accepted without additional discussion), “mutual agreement” (accepted in a modified form), “partially accepted” (completely accepted, but the information was lost while rewriting the therapy) or “not accepted”. Suggestions with no feedback were classified as “no information”.

### Ethical statement

The Institutional Medical Ethics Committee approved the study (UKC-MB-KME-27/19) and waived the need for informed consent because of the retrospective nature of the study. We conducted the study according to good clinical practice and protected the patients’ personal data according to the Law on Personal Data Protection.

### Statistical analysis

We performed only descriptive statistical analysis using IBM SPSS Statistics for Windows, Version 24.0. (IBM Corp., Armonk, NY, USA) after data were collected by Microsoft^®^Office Excel. The results were presented as numbers and percentages of the CP’s interventions in each intervention category and numbers and percentages of accepted recommendations.

## RESULTS

During the study period, 533 patients were admitted to the medical ICU. The CP reviewed prescribed medications of 321 patients and made 307 interventions in 95 patients. From all 307 interventions, 203 (66%) were related to antimicrobial drugs.

**FIGURE 1:**
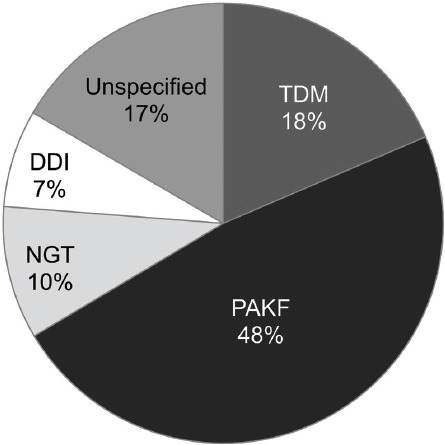
Distribution of the clinical pharmacist’s interventions (307 interventions) by intervention category. The PAKF category presented almost half of all interventions. PAKF: Pharmacotherapy adjustments to kidney function category; NGT: Nasogastric tube category; TDM: Therapeutic drug monitoring category; DDI: Drug-drug interaction category.

The distribution of the CP’s interventions according to the intervention category is presented in Figure 1.

There were 147 interventions of the PAKF category [in 58 patients] (Table 1). Almost one-third of interventions (27%) were associated with dose adjustments related to renal replacement therapy (RRT).

**TABLE 1:**
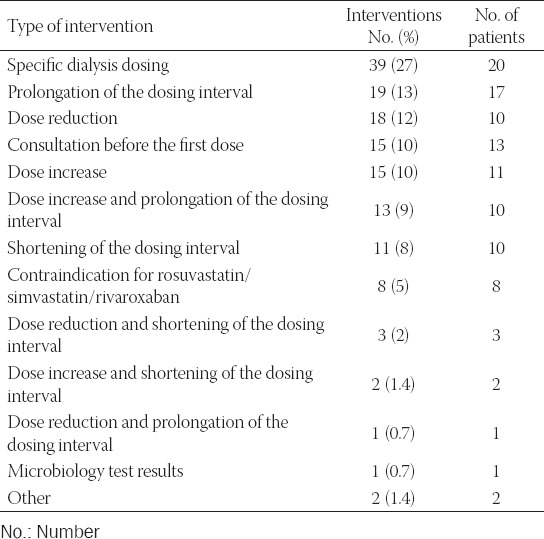
Clinical pharmacist’s interventions of the PAKF category (Pharmacotherapy adjustments to kidney function category) by type (147 interventions)

Dose adjustments to kidney function were most frequently suggested for antimicrobial drugs (124 interventions; 84% of the interventions of the PAKF category), mostly for meropenem, piperacillin/tazobactam, imipenem/cilastatin, cefepime, ciprofloxacin, and acyclovir. Non-antimicrobial drugs excreted by the kidneys represented 16% of the PAKF category (23 interventions), mainly levetiracetam, rosuvastatin, and methylnaltrexone. The CP suggested prescription of an equipotent dose of atorvastatin instead of rosuvastatin or simvastatin in seven cases, and low molecular weight heparin instead of rivaroxaban in one case.

There were 30 interventions regarding administration by the NGT, i.e., NGT category (in 20 patients), including seven interventions of replacing immediate-release crushed tablets with a suspension or liquid form due to easier administration and 23 interventions against the administration of the prescribed drugs in a crushed form through the NGT (Table 2).

**TABLE 2:**
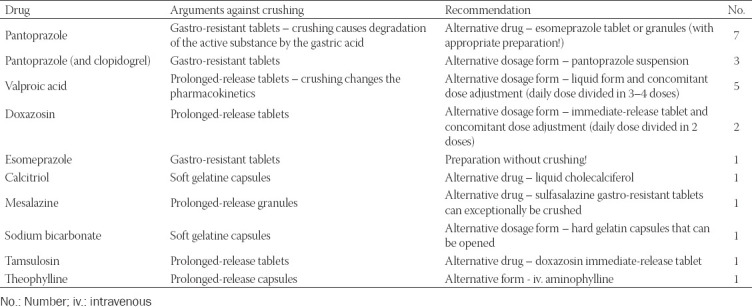
Clinical pharmacist’s interventions suggesting against the administration of prescribed drugs in a crushed form through the nasogastric tube (23 interventions)

The CP intervened 22 times within the DDI category [in 13 patients] (Table 3), most often due to concomitant use of clopidogrel and esomeprazole.

**TABLE 3:**
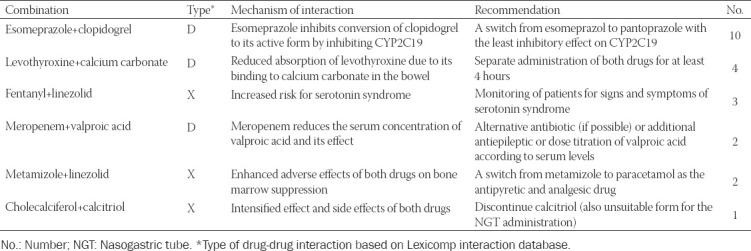
Clinical pharmacist’s interventions of the DDI category (drug-drug interaction category) (22 interventions)

The CP made 57 interventions of the TDM category (15 patients), including 38 interventions of vancomycin dosing, 15 interventions of amikacin dosing, three of gentamicin dosing, and one intervention of cyclosporine dosing.

There were 51 interventions of the Unspecified category [in 39 patients] (Table 4).

**TABLE 4:**
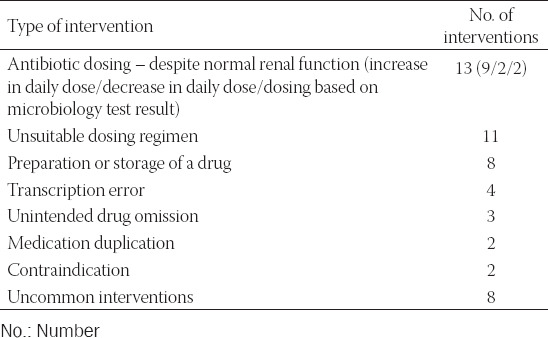
Clinical pharmacist’s interventions of the Unspecified category (51 interventions)

**FIGURE 2:**
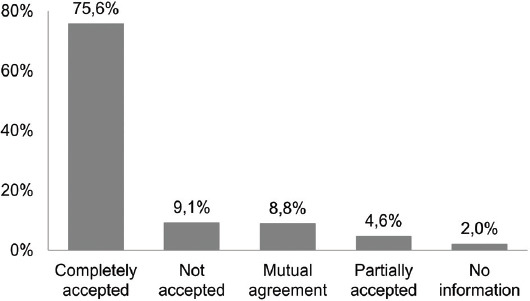
Acceptance of the clinical pharmacist’s (CP) recommendations by intensive care unit (ICU) physicians (307 interventions). The ICU physicians completely accepted 80.2% of the CP’s suggestions (Completely accepted plus Partially accepted); however, for 4.6% interventions, the information was lost while rewriting the therapy (Partially accepted). In addition, 8.8% suggestions were accepted after mutual agreement.

**FIGURE 3:**
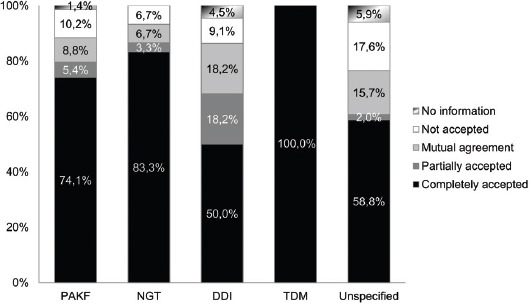
Acceptance of the clinical pharmacist’s recommendations by intensive care unit (ICU) physicians per intervention category. The majority of completely accepted suggestions were of the TDM category, as the pharmacist-guided TDM was the most familiar to the ICU physicians due to the previous cooperation with pharmacists. PAKF: Pharmacotherapy adjustments to kidney function category; NGT: Nasogastric tube category; TDM: Therapeutic drug monitoring category; DDI: Drug-drug interaction category.

The ICU physicians completely accepted 80.2% of the CP’s suggestions. However, for 4.6% interventions, the information was lost while transcribing the therapy. Figures 2 and 3 represent the acceptance of recommendations by the ICU physicians and the percentage of accepted recommendations per intervention category, respectively.

## DISCUSSION

In this retrospective analysis of the CP activities in the medical ICU team, we observed that regular participation of the CP contributed to more individualized and improved pharmacological treatment of patients. Our observations confirm that even a part-time presence of a CP in the ICU team intercepted a number of prescribing errors, having the potential for substantial clinical impact.

The intensity of CPs’ activities may differ among ICUs. In the study by Klopotowska et al. [2], two pharmacists reviewed the records of 1173 patients in a 28-bed medical/surgical adult ICU over 8.5 months and performed 659 interventions – that is 0.6 interventions per reviewed patient or 2.8 interventions per ICU bed per month. On the other hand, Johansen et al. [3] reported that three CPs reviewed the records of 363 patients in a 10-bed mixed adult ICU over 12 months and made 641 interventions. This represents 1.8 interventions per patient or 5.3 interventions per ICU bed per month. The study of Bourne et al. [31] reported that eight pharmacists, with an average of 5.4 years of critical-care experience, made 14.2 interventions per ICU bed per month in a general 22-bed ICU. During their study, two to three pharmacists were present in ward for 8 hours on weekdays and one pharmacist every Saturday. In our study, a single CP reviewed the records of 321 patients over a 9 month period and suggested 307 interventions in 95 patients, accounting for 1.0 intervention per patient or 2.8 interventions per ICU bed per month. It is worth mentioning again that the CP was present only part-time in our medical ICU. However, the education of the ICU team by the CP was another important aspect. While suggesting changes and explaining the background of pharmacotherapy, the CP educated the ICU staff regarding dosing, the route of administration, and the type of drugs to be used also in the absence of the CP.

In our study, almost half of all interventions were of the PAKF category. The majority of drug dose adjustments to kidney function were observed with prescription of antimicrobials. This was also observed in the studies of Jiang et al. [32] and Kim et al. [33]. Almost a third of interventions of the PAKF category was associated with dose adjustments related to RRT. As demonstrated by Jiang et al. [34], continuous RRT is most frequently associated with dosing errors, in particular during interruptions, modifications, and discontinuation of RRT. Prompt adjustments of drug dosing are therefore very important.

Inappropriate administration of medications by the NGT led to 23 interventions. Crushed pantoprazole gastro-resistant tablets were most often the subject of the CP’s intervention, and this was also observed in other studies [35,36]. The CP suggested to avoid the administration of large particles and crushed extended-release or gastro-resistant dosage forms by the NGT.

Interventions of the DDI category represented 7% of all interventions. However, potential DDIs can be identified in almost all of ICU patients. Smithburger et al. [37] identified 457 potential interactions in 240 ICU patients, and 25% of DDIs were considered as major (based on the Lexicomp^®^ and Micromedex^®^ databases). Ziehl et al. [38] identified potential DDIs in 96% of the studied patients, with only 5% of DDIs confirmed by the intensivists. The problem in identifying DDIs is the discrepancy in the rating of DDIs when different databases are used.

The well-established pharmacist-guided TDM at our institution was even improved in this study. Occasionally, the CP observed a delay in drug application as the result of diagnostic or treatment procedures or inappropriate timing of blood sampling. These factors are important in the daily practice of an ICU and should be taken into account when the dosing regimen is adjusted based on serum drug concentration.

We identified 51 interventions in the Unspecified category. Among them, 20 interventions were the result of human error and eight were associated with inappropriate preparation and storage of medication. In future, the CP should focus more intensely on preparation of parenteral drugs.

In the ICU team, there was a high rate of acceptance of the CP’s suggestions, as 80.2% of the suggestions were completely accepted and 8.8% were accepted after mutual agreement. In other studies, a 74% “rate of consensus” between pharmacists and physicians was found by Klopotowska et al. [2], 87% of the pharmacist’s suggestions were “accepted or taken into consideration” in the study of Johansen et al. [3], and 90% of “recommendation acceptance rate” was found by Bourne et al. [31]. In our study, the majority of completely accepted suggestions were of the TDM category, as the pharmacist-guided TDM was the most familiar to the ICU physicians due to previous successful cooperation with pharmacists.

Our study has several limitations. First, the study was a retrospective analysis of a CP’s interventions. Only a prospective study of a larger cohort of critically ill patients with additional evaluation of clinical outcomes would reveal the actual impact of the CP’s interventions. Second, we did not assess the clinical relevance of the CP’s interventions or the severity of the detected prescribing errors (like the assessment scale in Overhage and Lukes [39]). Third, only one CP participated part-time in the medical ICU team.

## CONCLUSION

We conclude that, despite the limitations of this retrospective analysis, the results of a single CP that collaborated with our medical ICU team only part-time are encouraging. A significant number of CP activities identified and prevented drug prescription errors and significant drug interactions, improved drug preparation and drug administration, individualized drug dosage, and decreased the risk of adverse events. The CP mainly intervened in four types of drug-related problems, such as pharmacotherapy adjustment to kidney function, drug-drug interactions, dosing adjustments of drugs with a narrow therapeutic index, and drug administration by the NGT. About 48% of all interventions were related to pharmacotherapy adjustment to kidney function and 66% of all interventions were associated with antimicrobial drugs. A dedicated CP in the ICU team improved the quality and safety of pharmacotherapy in critically ill patients.

## References

[1] Chant C, Dewhurst NF, Friedrich JO (2015). Do we need a pharmacist in the ICU?. Intensive Care Med.

[2] Klopotowska JE, Kuiper R, van Kan HJ, de Pont AC, Dijkgraaf MG, Lie-A-Huen L (2010). On-ward participation of a hospital pharmacist in a Dutch intensive care unit reduces prescribing errors and related patient harm:An intervention study. Crit Care.

[3] Johansen ET, Haustreis SM, Mowinckel AS, Ytrebø LM (2016). Effects of implementing a clinical pharmacist service in a mixed Norwegian ICU. Eur J Hosp Pharm.

[4] Malfará M, Pernassi M, Aragon D, Carlotti A (2018). Impact of the clinical pharmacist interventions on prevention of pharmacotherapy related problems in the paediatric intensive care unit. Int J Clin Pharm.

[5] Hisham M, Sivakumar MN, Veerasekar G (2016). Impact of clinical pharmacist in an Indian intensive care unit. Indian J Crit Care Med.

[6] Luisetto M, Mashori GR (2017). Intensive care units (ICU):The clinical pharmacist role to improve clinical outcomes and reduce mortality rate - an undeniable function. J Clin Intensive Care Med.

[7] Jacobi J (2016). Clinical pharmacists:Practitioners who are essential members of your clinical care team. Rev Méd Clín Cond.

[8] Borthwick M (2019). The role of the pharmacist in the intensive care unit. J Intensive Care Soc.

[9] Brilli RJ, Spevetz A, Branson RD, Campbell GM, Cohen H, Dasta JF (2001). Critical care delivery in the intensive care unit:Defining clinical roles and the best practice model. Crit Care Med.

[10] SmPC of drugs from National Drug Database.

[11] Up To Date ^®^ Database.

[12] Micromedex^®^ Database.

[13] Ashley C, Currie A (2009). The Renal Drug Handbook.

[14] Erstad B (2016). Critical Care Pharmacotherapy.

[15] (2017). Stanford Hospital and Clinics Antimicrobial Dosing Reference Guide.

[16] CRRT Antimicrobial Dosing Recommendation, University of Pennsylvania Health System (2015). Department of Pharmacy;. http://www.uphs.upenn.edu/surgery/education/trauma/sccs/protocols/crrt_antimicrobial_dosing_table.pdf.

[17] White R, Bradnam V (2015). Handbook of Drug Administration via Enteral Feeding Tubes.

[18] Wang RZ, Vashistha V, Kaur S, Houchens NW (2016). Serotonin syndrome:Preventing, recognizing, and treating it. Cleve Clin J Med.

[19] van Ewijk CE, Jacobs GE, Girbes AR (2016). Unsuspected serotonin toxicity in the ICU. Ann Intensive Care.

[20] Pedavally S, Fugate JE, Rabinstein AA (2014). Serotonin syndrome in the intensive care unit:Clinical presentations and precipitating medications. Neurocrit Care.

[21] Bouziana SD, Tziomalos K (2015). Clinical relevance of clopidogrel-proton pump inhibitors interaction. World J Gastrointest Pharmacol Ther.

[22] Wedemeyer RS, Blume H (2014). Pharmacokinetic drug interaction profiles of proton pump inhibitors:An update. Drug Saf.

[23] Abraham NS, Hlatky MA, Antman EM, Bhatt DL, Bjorkman DJ, Clark CB (2010). ACCF/ACG/AHA 2010 expert consensus document on the concomitant use of proton pump inhibitors and thienopyridines:A focused update of the ACCF/ACG/AHA 2008 expert consensus document on reducing the gastrointestinal risks of antiplatelet therapy and NSAID use. A report of the American college of cardiology foundation task force on expert consensus documents. J Am Coll Cardiol.

[24] (2010). FDA Reminder to Avoid Concomitant use of Plavix (Clopidogrel) and Omeprazole.

[25] (2009). EMA Public Statement on Possible Interaction Between Clopidogrel and Proton Pump Inhibitors.

[26] Lee MC, Sun YH, Lee CH, Wu AJ, Wu TW (2012). Interaction between valproic acid and carbapenems:Case series and literature review. Tzu Chi Med J.

[27] Mori H, Takahashi K, Mizutani T (2007). Interaction between valproic acid and carbapenem antibiotics. Drug Metab Rev.

[28] Spriet I, Goyens J, Meersseman W, Wilmer A, Willems L, Van Paesschen W (2007). Interaction between valproate and meropenem:A retrospective study. Ann Pharmacother.

[29] Huang CR, Lin CH, Hsiao SC, Chen NC, Tsai WC, Chen SD (2017). Drug interaction between valproic acid and carbapenems in patients with epileptic seizures. Kaohsiung J Med Sci.

[30] Bede P, Lawlor D, Solanki D, Delanty N (2017). Carbapenems and valproate:A consumptive relationship. Epilepsia Open.

[31] Bourne RS, Choo CL, Dorward BJ (2014). Proactive clinical pharmacist interventions in critical care:Effect of unit speciality and other factors. Int J Pharm Pract.

[32] Jiang SP, Chen J, Zhang XG, Lu XY, Zhao QW (2014). Implementation of pharmacists'interventions and assessment of medication errors in an intensive care unit of a Chinese tertiary hospital. Ther Clin Risk Manag.

[33] Kim AJ (2015). Pharmacotherapeutic problems and pharmacist interventions in a medical intensive care unit. Korean J Crit Care Med.

[34] Jiang SP, Zhu ZY, Ma KF, Zheng X, Lu XY (2013). Impact of pharmacist antimicrobial dosing adjustments in septic patients on continuous renal replacement therapy in an intensive care unit. Scand J Infect Dis.

[35] Induja L, Aslam TA, Chithra S, Andhuvan G (2018). Pharmacist intervention and preparation of manual in the administration of drugs through enteral feeding tube. Int J Pharm Pharm Sci.

[36] Sohrevardi SM, Jarahzadeh MH, Mirzaei E, Mirjalili M, Tafti AD, Heydari B (2017). Medication errors in patients with enteral feeding tubes in the intensive care unit. J Res Pharm Pract.

[37] Smithburger PL, Kane-Gill SL, Seybert AL (2012). Drug-drug interactions in the medical intensive care unit:An assessment of frequency, severity and the medications involved. Int J Pharm Pract.

[38] Ziehl EA, Morales FE, Villa LA (2019). Drug-drug interactions in an intensive care unit of a tertiary hospital in southern Chile:Evaluating databases agreement. J Pharm Pharmacogn Res.

[39] Overhage JM, Lukes A (1999). Practical, reliable, comprehensive method for characterizing pharmacists'clinical activities. Am J Health Syst Pharm.

